# Evolution and surgical management of a *Mycobacterium avium* complex aortic infection

**DOI:** 10.1016/j.jvscit.2025.101763

**Published:** 2025-02-26

**Authors:** Fletcher N. Pierce, Gear T. Vincent, Aubrey Mwinyogle, Jean M. Panneton

**Affiliations:** Division of Vascular Surgery, Eastern Virginia Medical School, Norfolk, VA

**Keywords:** Mycobacterium avium, Thoracoabdominal aortic aneurysm (TAAA), Infrarenal aortic aneurysm, Endovascular aortic repair (EVAR), AIDS, Aortic infection

## Abstract

We present a case describing the successful surgical management of both an infected aneurysm and an aortic graft infection in a patient with disseminated *Mycobacterium avium* complex infection and AIDS. The patient was a 53-year-old man presenting with a ruptured type V thoracoabdominal aortic aneurysm and an isolated 7.1-cm infrarenal abdominal aortic aneurysm. He underwent emergent repair of his ruptured thoracoabdominal aneurysm using a rifampin-impregnated Dacron graft. This was followed by a temporizing endovascular aortic repair and was eventually bridged with an in situ repair using a cryopreserved cadaveric aortic homograft.

Infected aneurysms and aortic graft infections are surgical challenges often mandating open repair and debridement of infected tissue or prosthesis. Common culprits of such aortic infection include *Staphylococcus aureus*, *Salmonella* species, *Pseudomonas aeruginosa*, and polymicrobial anaerobes. Although there are occasional reports of tuberculosis-associated aortic infections, reports of *Mycobacterium avium* complex (MAC)-infected aneurysms and aortic graft infection are rare.

We present a case of successful surgical treatment of a contained rupture of an infected thoracoabdominal aortic aneurysm (TAAA) followed by explantation of an infected endovascular aortic repair (EVAR) endograft in the setting of MAC aortic infection in a patient with AIDS.

## Case report

**Fig 1:**
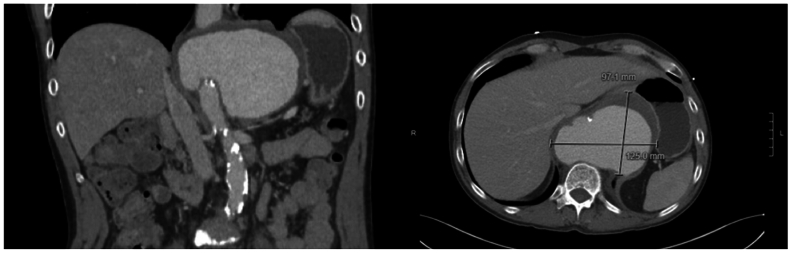
Contained rupture of a type V thoracoabdominal aneurysm with aneurysm sac measuring 9.7 × 12.5 cm with mass effect on the distal esophagus and diaphragmatic crus partially narrowing the origin of the celiac and 50% to 70% narrowing of the superior mesenteric artery.

**Fig 2:**
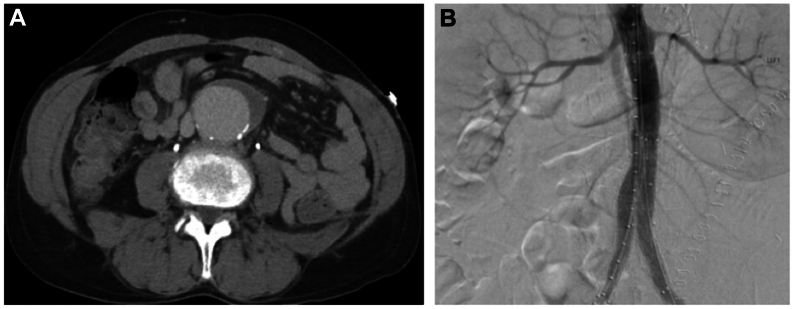
**(A)** Isolated 7.1 × 4.3 × 5.0 cm infrarenal aneurysm extending to the bifurcation. **(B)** Completion angiogram after endovascular aortic repair (EVAR).

A 53-year-old man with a past medical history of AIDS on highly active antiretroviral therapy with a CD4 count of 153, hypertension, and a 30 pack-year smoking history presented to an outside hospital with angina. He was found to have a contained rupture of a type V TAAA (Fig 1) and an isolated 7.1-cm infrarenal abdominal aortic aneurysm (AAA) (Fig 2, *A*). He was emergently air transferred to our facility. We performed an emergent open repair of his thoracoabdominal aneurysm using a 28-mm rifampin-impregnated Dacron graft (RIDG) with a beveled distal anastomosis to include the celiac and superior mesenteric artery origins.

Given his history, there was high suspicion for infection. Aortic tissue and thrombus were sent for culture and pathology. Gram stain and pathology were negative; however, cultures grew acid-fast bacilli, MAC, on postoperative day 15.

On postoperative day 17, the patient experienced excruciating abdominal pain and there was concern for impending AAA rupture. However, after his open thoracoabdominal repair, he had developed transient respiratory failure and acute kidney injury with a peak serum creatinine of 3.8. Given his deconditioned state, we proceeded with a temporary EVAR with a 23 × 14 × 104 mm polyester endograft with bilateral iliac extensions. To mitigate infection risk, all stent graft materials were injected with rifampin. A completion angiogram demonstrated no evidence of endoleak (Fig 2, *B*). He was pain free and breathing room air, with a down-trending creatinine of 1.9. He was discharged post-EVAR day 2. In coordination with infectious disease (ID), he was discharged on antiviral therapy, daptomycin, ceftriaxone, rifampin, ethambutol, and azithromycin. We planned follow-up for 1 month later to arrange explantation of the endograft.

**Fig 3:**
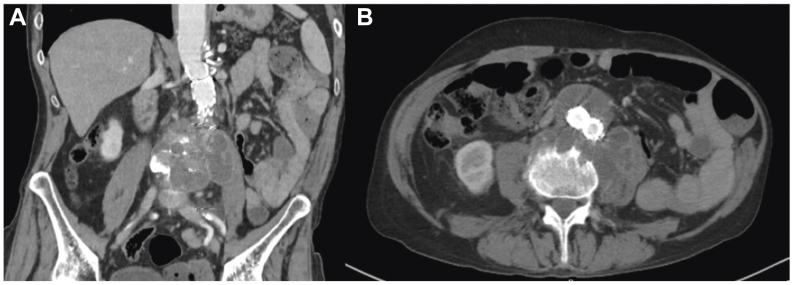
**(A** and **B)** Computed tomography angiography (CTA) revealing a 7.5 × 6.0 cm loculated periaortic fluid collection eroding L3-L4 vertebrae and extending down the left psoas muscle.

He was absent from follow-up and presented 3 years later with back pain after a fall from standing. His CD4 count had improved from 153 to 226 cells/mm^3^. Computed tomography angiography (CTA) revealed a 7.5 × 6.0 cm loculated periaortic fluid collection eroding L3-L4 vertebrae and extending down the left psoas muscle (Fig 3). He was started on broad spectrum antibiotics. A drain was placed under CT guidance and tissue aspirate was sent for culture. Again, cultures grew acid-fast bacilli (MAC).

**Fig 4:**
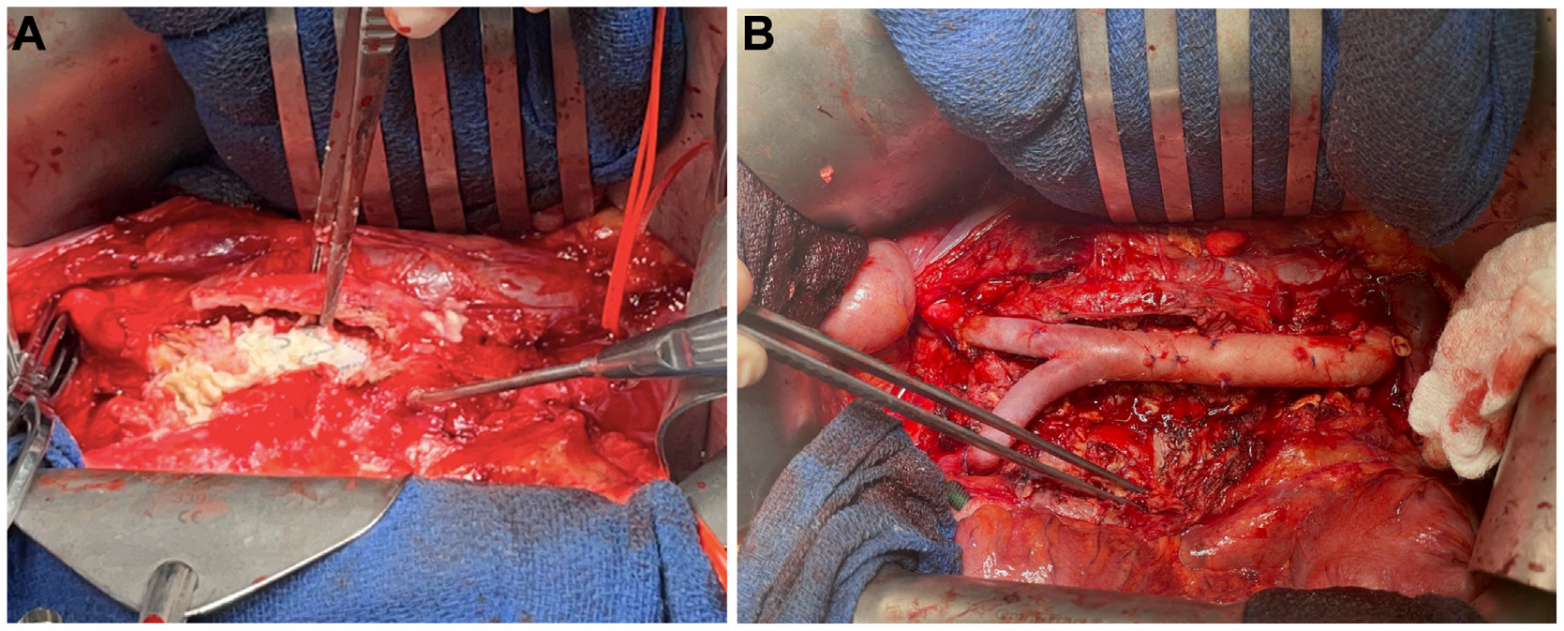
**(A)** Exposure of the infected endograft. **(B)** In situ repair using a cryopreserved cadaveric aortic homograft before being wrapped in a pedicled omental flap.

The patient was restarted on antimycobacterial antibiotics before surgery. With a proximal suprarenal clamp, we explanted the infected endograft (Fig 4, *A*) using wire cutters to separate the suprarenal fixation stents. The aneurysm sac and surrounding tissues were thoroughly debrided and irrigated. Neurosurgery declined further debridement of the vertebrae. We then performed in situ repair using a cryopreserved cadaveric aortoiliac homograft with distal anastomoses to both iliac arteries (Fig 4, *B*). Finally, a pedicled omental flap was wrapped circumferentially around the graft.

He was discharged on postoperative day 7 with continued antimycobacterial antibiotics, vancomycin, meropenem, and antiretroviral therapy with planned routine follow-up with ID. Our vascular surgery department arranged follow-up with CTA surveillance at 1, 3 and 6 months postoperatively, with subsequent imaging yearly. The patient has not returned for CTA follow-up to date. However, he has been following with ID and was reported to have been doing well 6 months after his open AAA repair.

## Discussion

HIV/AIDS is associated with an increased risk of AAA and infected aneurysms.^1^ In patients with AIDS, MAC typically presents as disseminated disease or lymphadenitis, although disseminated MAC is rare in patients with HIV on highly active antiretroviral therapy. Only five known additional reported cases of MAC causing infected aneurysms were observed upon literature review.^2^, ^3^, ^4^, ^5^, ^6^ Four of these five patients had HIV and only one patient was reported to have a CD4 count of less than 200.

Ziehl-Neelsen stain and pathology help with rapid identification of MAC. Additionally, DNA probes are used to distinguish between MAC and tuberculosis when an acid-fast bacillus is isolated. Treatment of MAC includes a macrolide and ethambutol with or without rifabutin, because monotherapy conveys a high rate of macrolide resistance.^7^

Surgical management of MAC-infected aneurysms follows the principles of any infected aneurysm repair and requires long durations of antibiotics. Options for revascularization after resection and debridement of an infected aortic aneurysm or graft include extra-anatomic axillary-bifemoral bypass or in-situ repair with either a RIDG, a cryopreserved allograft, or a neoaortoiliac system (NAIS). In situ repair with aortic homograft or RIDG offers superior freedom from thrombosis, anastomosis breakdown, and adverse limb events when compared with extra-anatomical bypass.^8^, ^9^, ^10^, ^11^ The NAIS procedure involves femoral vein and/or popliteal vein harvest for use as a conduit. This technique has demonstrated a low risk of reinfection. NAIS is also associated with long operative times and the possibility of venous compartment syndrome and chronic venous insufficiency.^11^ In an emergent setting, such as when this patient initially presented with a contained rupture of a type V TAAA, cryopreserved allografts and the NAIS will not be available expeditiously and RIDG offers excellent patency with acceptable resistance to reinfection.

In certain circumstances, RIDGs can be used as a last resort bridging therapy for large infected aneurysms in patients unfit for immediate open repair.^12^^,^^13^ For endografts to retain the rifampin coating effectively, they must be made of polyester rather than PTFE. Currently, there are no polyester US Food and Drug Administration-approved EVAR devices without suprarenal fixations. Therefore, in the setting of primary aortic infection or infection of the RIDG itself, surgically explanting the endograft requires removal of the suprarenal stent. This can be achieved using a 20-mL syringe with the top cut off to recapture the fixation struts or using oral maxillofacial surgery wire cutters, as was performed in this case.^14^

With the patient stable upon re-presentation, cryopreserved allograft was deemed suitable for repair of this patient's infected abdominal aortic endograft owing to excellent freedom from infection and similar technical results as a RIDG. Of note, early cryopreserved allografts were prone to anastomotic breakdown and continued aneurysmal degeneration; however, more recent products demonstrate lower incidence of such complications.^10^ Finally, after completion of the repair, a pedicled omental flap was dissected and secured circumferentially around the cryograft to decrease incidence of reinfection and aortoenteric fistula.

The patient was discharged on ethambutol, rifabutin, and azithromycin for MAC coverage, as well as vancomycin and meropenem for typical bacterial pathogens. Plans were established for life-long CTA surveillance and suppressive antibiotics managed in coordination with ID.

## Conclusions

In situ repair with aortic homograft or RIDG offers superior freedom from reinfection, thrombosis, anastomosis breakdown, and adverse limb events when compared with extra-anatomical bypass. Additionally, RIDGs can be used as a bridging therapy for infected aneurysms in patients ineligible for open repair. This case demonstrates the successful treatment of an infected aneurysm using bridging endovascular repair and an aortic graft infection in a patient with disseminated MAC infection and AIDS.

## Funding

None.

## Disclosures

JMP reports relationships with Terumo Aortic, Medtronic Inc., Getinge, W. L, Gore & Associates, Penumbra, and Philips.
